# iEthics: An Interprofessional Ethics Curriculum

**DOI:** 10.3390/pharmacy10010012

**Published:** 2022-01-06

**Authors:** Victoria Wood, Lynda Eccott, Philip Crowell

**Affiliations:** 1 Office of the Vice-President, Health, The University of British Columbia, 400-2194 Health Sciences Mall, Vancouver, BC V6T 1Z3, Canada; 2 Faculty of Pharmaceutical Sciences, The University of British Columbia, 2405 Wesbrook Mall, Vancouver, BC V6T 1Z3, Canada; lynda.eccott@ubc.ca; 3 Faculty of Medicine, The University of British Columbia, 4500 Oak Street, Vancouver, BC V6H 3N1, Canada; PCrowell@cw.bc.ca

**Keywords:** interprofessional education, ethics, case-based learning, program evaluation, blended learning

## Abstract

This article discusses the development, content, implementation, and evaluation of an interprofessional ethics curriculum that has been integrated as a required component of learning in the Faculty of Pharmaceutical Sciences at the University of British Columbia (UBC), along with 12 other health professional programs. We start by giving a background and rationale for the development of the integrated ethics (iEthics) curriculum, led by UBC Health, and provide an overview of the pedagogical approach used, curriculum model, and content. We outline the way in which the iEthics curriculum has been implemented in the Faculty and share findings from program evaluations. In the discussion section, we reflect on our experience as facilitators for the interprofessional workshops and link these experiences with the findings from the program evaluations. These reflections highlight the way in which the iEthics curriculum has been successful in meeting the desired outcomes of learning in terms of the interprofessional delivery, and provide insights into how the findings from the iEthics evaluation informed other modules in the integrated curriculum and its implementation in the Faculty of Pharmaceutical Sciences.

## 1. Introduction

Ethics is an essential component of all health professional education [1]. Believing that learning about ethics could be enhanced by an interprofessional approach [2], a group at the University of British Columbia (UBC) came together to develop an interprofessional ethics curriculum. As had been done with similar topics, such as pain management, we identified ethics as a topic that could act as a vector for interprofessional learning [3]. Like comparable initiatives, we found that there was common content related to ethics across the health disciplines at UBC, providing a significant opportunity for economies of scale in terms of the delivery of this content across programs [4,5].

In practice, ethical issues also transcend disciplinary boundaries, as many can only be adequately addressed using an interprofessional collaborative approach. Resolving ethical dilemmas requires interdisciplinary relationships and collaboration [6]. As such, using an interprofessional approach to teaching ethics, in addition to other complex areas of healthcare, has been shown to greatly enhance student learning and better prepare them to work within existing and emerging models of team-based care [4].

Interprofessional education (IPE) is being integrated into health professional programs across the world, including at UBC, as a mechanism to build the collaborative competencies that support team-based care [7]. A university-based unit (UBC Health), with the specific mandate to foster and support collaborative practice through the development and implementation of IPE, brought together an interprofessional working group of content experts, educators, learners, and patients/clients to develop an integrated ethics (iEthics) curriculum. Led by a faculty member from the Faculty of Pharmaceutical Sciences, the goal was to develop an innovative approach to ethics education by using a pedagogy that was interprofessional, case-based, values-focused and interactive.

Previously, students in the Faculty of Pharmaceutical Sciences PharmD program, and other participating programs, engaged in uni-disciplinary learning about ethics. Students in Pharmacy participated in an introductory lecture on professionalism and ethics, focusing on professional boundaries and ethical decision-making in a uni-professional setting. In order to explore the viability of developing an integrated ethics curriculum, an environmental scan of existing competencies, learning objectives and ethics content across the health disciplines at UBC was used to identify opportunities for economies of scale related to common content and areas that would benefit from a collaborative approach, resulting in the iEthics modules discussed in this paper.

This article provides an overview of the development, content, and implementation of the iEthics Curriculum that was delivered at UBC between 2017 and 2019. We share findings from program evaluations, and in the discussion section we link these findings to reflections from our experiences facilitating the interprofessional sessions over a three-year period. These reflections highlight the ways in which the curriculum has been able to meet the desired outcomes of learning in terms of the interprofessional delivery. Finally, we discuss how these evaluations influenced the ongoing integration of the iEthics modules into the broader Faculty of Pharmaceutical Sciences curriculum. The discussion section also provides insights into how the finding from the iEthics evaluations fed into the broader integrated curriculum created by UBC Health and subsequent modules.

## 2. Materials and Methods

This project involved the development, integration, and evaluation of an interprofessional ethics curriculum.

### 2.1. Development

An interprofessional working group, comprised of faculty, staff, students, and community members with expertise in ethics and/or interprofessional education began this process by developing a set of guiding principles and an interprofessional ethics competency framework, both of which provided a foundation and grounding for the integrated content. These were based on a mapping of ethics competencies and content from across the 13 participating health professional programs [4]—Audiology, Dental Hygiene, Dentistry, Dietetics, Genetic Counseling, Medicine, Midwifery, Nursing, Occupational Therapy, Pharmacy, Physical Therapy, Social Work, and Speech Language Pathology.

Given the differing lengths of participating programs, a modular approach ensured flexibility, with online modules delivering foundational content in preparation for interactive, interprofessional workshops. This facilitated the integration of the curriculum within Pharmacy, a 4 year program, as well as into other programs that are completed in 2 years, for example, Physical Therapy. Each module built in complexity, with the first providing important content to ensure students from different professions had a common foundation for the discussion of a complex ethical case during the second workshop. The intent was to replace existing ethics content in the PharmD Program, and other participating programs, and enhance learning related to ethics through an interprofessional approach. Details about each module and workshop are shared below.

#### 2.1.1. Foundations of Ethical Practice (One-Hour Preparatory Online Module Followed by a Two-Hour Interprofessional Workshop)

During the online module, students considered ethical situations in everyday life, explored their personal and professional values, and were exposed to the role of professional codes of ethics and an ethical decision-making framework (EDMF). Students learned about the principles of health care ethics and the importance of ethical practice. During the interprofessional workshop, students came together to delve more deeply into issues related to ethical practice. Pharmacy students came together with a mix of students from other professions to compare their values and professional codes of ethics. In addition, they had the opportunity to discuss two low-complexity cases, the first centering around Mrs. S, a woman at high risk of a stroke, which focused on her cultural values, capability, and issues of consent. The second case had students consider relational autonomy within the context of a transplant case involving the J family. The module and workshop were designed to act as a foundation for more complex learning around ethical practice during the second interprofessional workshop. This module was completed in the first year of the PharmD program.

#### 2.1.2. Ethical Decision-Making (Two-Hour Self-Directed Online Module)

This online module was designed to be delivered after the Foundations of Ethical Practice module and workshop. During this module, students dove more deeply into the elements of the interprofessional ethical decision-making framework (EDMF) presented during the foundational module. After being exposed to an example of how the framework could be applied to a specific case, students worked through a case on their own. The example case, presented through a series of videos, introduced the patient and broke down the case using the EDMF, focusing on issues of informed consent and autonomy for Jason, a young hockey player whose healthcare decisions were being influenced by his coach. Students then used the EDMF to work through the case of Mrs. D, a woman living with dementia whose partner appeared to be suffering from caregiver burnout and may have become abusive. Again, the case was presented through a series of engaging videos. Pharmacy students completed this during the second year of their program.

#### 2.1.3. Interprofessional Ethical Decision-Making (Half-Hour Self-Directed Online Module Followed by a Two-Hour Interprofessional Workshop)

The first module and workshop provided a strong foundation for this second interprofessional workshop, during which students came together with learners from other professions for an interactive, case-based discussion centered on a complex ethical case. This case focused on end of life options and Medical Assistance in Dying (MAID), which was chosen because of the timeliness and complexity of this issue, having recently been legalized in Canada. A short online preparatory module provided an overview of MAID. The case introduced Karla, a young woman who had requested MAID when diagnosed with Stage 4 metastasized cancer after undergoing treatment for Stage 3 cervical cancer a few years earlier. The patient and family were brought together in a fictional case that was an amalgamation of real patients and experiences of the working group members. Having a young patient, with a complex family situation and same-sex partner, made the case relatable for students. The case, involving multiple professions, was introduced through a series of videos of the patient interacting with her healthcare team and family. The case was disclosed through ‘role cards’ that provided information from each of the participating professions. Students were provided with the following information from a pharmacist’s perspective:

“*I am concerned that Karla stopped taking her anti-depressants suddenly when she was re-diagnosed with cancer. Antidepressant Discontinuation Syndrome can result in flu-like symptoms and disturbances in sleep, senses, movement, mood, and thinking. I am also concerned that her pain is not being adequately managed. When I spoke with her recently, she complained about the severe pain in her back, despite being on morphine. From my perspective, it is important to talk to Karla about the medications that could mitigate many of the side effects should she choose chemo and radiation. There are some that have been newly developed since her last experience with treatment 3 years ago.*”

This and other information about Karla’s physical and emotional wellbeing promoted discussions about how the team could work collaboratively to support Karla, so she might make an informed decision about MAID. During the workshop, students acted as members of an interprofessional ethics committee and had to make recommendations to Karla’s healthcare team, providing a rationale for each. Once the students had completed the case, it was debriefed using a video of an ethics committee discussing the situation. This module and workshop were completed in year 3 of the PharmD program.

#### 2.1.4. Moral Distress in Ethical Practice (One-Hour Self-Directed Online Module to Support Student Learning around Ethical Practice While on Placement)

This online module was designed to consolidate learning related to interprofessional ethical practice, focusing on moral resilience. It introduced a 4-step approach to rising above moral distress put forth by the American Associate of Critical-Care Nurses [8]. The module was designed to support learners to work through a guided reflection based on their experience in practice. This module was optional for Pharmacy students.

### 2.2. Integration and Evaluation

The development of the iEthics curriculum was an iterative process that took place over a three-year period, starting in 2014. Each module and workshop was developed, piloted, and revised before being integrated as a required component of the health professional programs at UBC. The iEthics curriculum was delivered in its entirety between 2017 and 2019, with significant changes in 2020 and 2021 not described here to accommodate restrictions to in-person learning during the COVID-19 pandemic. Between 2017 and 2019, the two interprofessional iEthics workshops discussed in this paper were delivered in-person in October and November to over 1000 students from 13 health professional programs. Learners completed the preparatory online modules during the two weeks preceding each workshop.

Learners were assigned to rooms with 40–60 students and were then placed in interprofessional groups of six during each two-hour workshop. The first and second workshop had different disciplinary mixes, depending on the programs available at that time. First year Pharmacy students participating in the first interprofessional workshop were grouped with students from Audiology, Dietetics, Physical Therapy, Occupational Therapy, Social Work, and Speech Language Pathology. The disciplinary mix for the second interprofessional workshop with the third-year Pharmacy students included Medicine, Midwifery, and Nursing. This gave the Pharmacy students an opportunity to interact with a wide range of disciplines. Each room had 1–2 facilitators who were provided with training to deliver the sessions.

After each session, participating students were emailed an online evaluation survey asking them open-ended qualitative questions about what they liked and what they would like to see improved, and quantitative questions about whether they felt the learning objectives were achieved using a 5-point Likert scale (strongly agree; agree; neutral; disagree; strongly disagree). They were also given the opportunity to provide qualitative comments explaining their choices about the learning objectives. A thematic analysis of the qualitative comments was conducted by two research assistants and then compared to identify key themes [9]. Ethics approval was not required for this project at it fell under the UBC BREB exception of program evaluation.

## 3. Results

The iEthics curriculum was integrated as a required component of student learning across the following 13 health professional programs at UBC—Audiology, Dental Hygiene, Dentistry, Dietetics, Genetic Counseling, Medicine, Midwifery, Nursing, Occupational Therapy, Pharmacy, Physical Therapy, Social Work, and Speech Language Pathology. Between 2017 and 2019 we received 1242 surveys from respondents representing all 13 programs. Quantitative survey findings, summarized in Table 1 and Table 2 indicate that the workshops were effective in achieving the desired learning objectives related to both the ethics content and the interprofessional learning.

The thematic analysis of qualitative comments indicated overwhelmingly that what students valued most were the case-based and interprofessional approaches to learning. Specifically, the themes that emerged were: (1) the value of case-based learning; (2) the value of the interprofessional approach; and (3) the value of complexity. Student comments talked about how videos enhanced the case and how ethics as a content area was enhanced by the interprofessional approach. This supported the quantitative results, which revealed that over 83% of respondents across all three years felt the workshops were enhanced by the interprofessional approach. While students valued the case-based approach used in both interprofessional workshops, they appreciated the complexity of the case used in the second workshop that focused on the complex issue of MAID. The following comments from students representing different disciplines exemplify the themes that emerged through the thematic analysis:

“*The case was very interesting and introduced a number of factors that we rarely discuss in our respective professions. I sincerely enjoyed the depth and care that was put into the case, including the additional perspectives from other healthcare providers*”.

(Pharmacy Student)

“*After talking to my colleagues, we all agreed, this was the best IPE session yet! The case was well designed, and had many intricacies to it. This allowed our group to form a great discussion. I also thought that the information points from each of the other specialties was a nice add on!*”

(Medical Student)

“*I liked getting to meet students from other health disciplines, and understand their role in the health setting and how we all kind of fit and work together to promote the patient’s care and well-being*”.

(Nursing Student)

“*The end-of-life videos were done very well. Many different perspectives were taken into account, the situation was quite realistic and heart wrenching. The video actors were all amazing and the excellent video really set the room for excellent discussions*”.

(Dentistry Student)

These and other qualitative comments from respondents stress the value of the interprofessional approach to the delivery. There were some comments related to the topic of ethics that suggest students felt that their uni-disciplinary content addressed the learning objectives related to ethics better than the interprofessional sessions.

“*The question asks if this was a result of this session. I feel like I already got this stuff about ethics in my program and was able to bring this into this session*”.

(Pharmacy Student)

These types of comments suggest that the interprofessional workshops were less effective in achieving the desired outcomes related to ethics.

## 4. Discussion

The evaluations indicate that ethics acted as an effective vector for interprofessional learning and that learning about ethics was enhanced by the interprofessional approach, as has been found with other content areas such as pain management [3]. Similar studies evaluating interprofessional ethics education have found that interprofessional teams bring out perspectives that do not emerge during uni-disciplinary settings [2]. As we facilitated the interprofessional sessions within the iEthics curriculum, we noticed how having students from different programs in the same session served to improve the level of respect for differences in personal and professional values and perspectives. There was a notable difference in the tone of discussions when students were in interprofessional groups. Students demonstrated an openness and curiosity during these sessions that was not always present during uni-disciplinary sessions that we have facilitated. Within an interprofessional context, students seemed to focus on their common commitment to patient-centered care, bringing together their different professional roles and perspectives around a common goal. They talked about relationship-based care, who was best positioned to serve the needs of the patient and their families, and what other professions should be involved in their care.

While students valued the interprofessional approach above all else, our evaluations of the iEthics curriculum also provide evidence that ethics, as the topic, offered a level of complexity that engaged students interprofessionally in meaningful ways. Our findings from the iEthics evaluations over three years support findings from similar program evaluations which indicate that it is important to provide students with opportunities to explore their own personal and professional values in relation to students from other professions [4]. From our experience facilitating the iEthics workshops, it was critical and interesting to see different ethical options that emerged from the face-to-face encounters with other students from different disciplines, and how some small but significant factors in terms of information or nuances in approaches to communication with the patients could shift the students’ conversations, dialogue, and discussions in a way that did not happen in uni-disciplinary learning sessions. Like Naidoo et al. [4], we saw how students valued the opportunity to explore their codes of ethics and develop an understanding of how to use an ethical decision-making framework in an interprofessional setting. Additionally, we learned that students appreciated learning about complex ethical issues through the professional roles and responsibilities of their interprofessional peers.

Based on these evaluations, both interprofessional workshops have been integrated into Pharmacy primarily as a means to provide interprofessional learning rather than as a way to deliver needed ethics content. Ethics is treated very much as a vector for interprofessional learning. The Faculty of Pharmaceutical Sciences has made the two workshops mandatory as a means for students to gain experience working as part of an interprofessional team. As such, the two standalone online modules that do not provide interprofessional learning, but instead were designed to provide additional content on the topic of ethics, have not been integrated as required learning in Pharmacy. However, there are a number of participating programs, such as Physical Therapy, that have integrated the entire iEthics curriculum in place of their uni-disciplinary ethics content. The iEthics curriculum has not replaced all uni-disciplinary content related to ethics in the Pharmacy program, with additional pharmacy-specific ethical content still being taught by the faculty. This uni-disciplinary content helps students develop a strong grounding in pharmacy perspectives in advance of their interprofessional learning.

### Study Limitations

There are several limitations to this program evaluation that warrant further study. Firstly, the evaluations relied on student perceptions about their learning, which might be better explored through approaches used by others, such as the use of ethical decision-making scores [1]. Secondly, it would have been interesting to conduct a cross-disciplinary comparison of student perceptions; however, we were unable to do so based on the small response rates within individual disciplines. Thirdly, the impact of integrating the entire iEthics curriculum versus certain sessions needs to be examined. Finally, further research is needed to assess the impact of this learning on future practice.

## 5. Conclusions

The iEthics Curriculum has been successful in supporting programs in meeting interprofessional accreditation standards; students in developing interprofessional competencies; and learning around ethics as a complex area of practice that benefits from a collaborative approach. Before the Integrated Curriculum, students in the Faculty of Pharmaceutical Sciences and other programs had limited exposure to learning through an interpersonal lens. To date, the iEthics curriculum has enhanced or replaced existing ethics content across 13 health professional programs at UBC, depending on the additional, unique disciplinary learning needs of students. As such, this interprofessional curricular model has been used as an exemplar for further interprofessional curricula at UBC, with other complex areas of healthcare that are common across disciplines acting as a vector for interprofessional learning, such as Professionalism, Health Informatics, Indigenous Cultural Safety, and Health Informatics.

## Figures and Tables

**Table 1:** Foundations of Ethical Practice Interprofessional Workshop—Student Perceptions about Learning Objectives Achieved.

Student Perceptions	Agreed or Strongly Agreed		
	2017 *n* = 280	2018 *n* = 187	2019 *n* = 90
Able to articulate how personal and professional values, beliefs and perspectives influence ethical decision making	82% (229)	84% (157)	84% (76)
Able to compare codes of ethics from different professions	85% (238)	84% (157)	84% (76)
Able to use professional code of ethics to describe professional responsibilities in relation to specific ethical scenarios	91% (255)	86% (161)	86% (77)
Able to describe how the fundamental elements of an ethical decision-making framework might be applied in specific cases	86% (241)	78% (146)	78% (70)

**Table 2:** Ethical Decision-Making Interprofessional Workshop—Student Perceptions about Learning Objectives Achieved.

Student Perceptions	Agreed or Strongly Agreed		
	2017 *n* = 279	2018 *n* = 327	2019 *n* = 79
Able to demonstrate how to effectively approach differences in their personal values and beliefs with those of others as they relate to ethical practice	80% (223)	70% (229)	80% (63)
Able to consider multiple perspectives in addition to their own when involved in shared ethical decision making	89% (248)	79% (258)	89% (70)
Able to demonstrate collaborative practice competencies with other members of the healthcare team when engaged in complex ethical discussions	87% (243)	79% (258)	87% (69)
Able to apply an ethical decision-making framework to a complex clinical situation in an interprofessional setting.	83% (231)	79% (258)	82% (65)

## Data Availability

The data presented in this study are available on request from the corresponding author.
